# CRYPTOMICSDB: Revealing the Molecular Landscape of Cryptococcosis

**DOI:** 10.3390/jof11060425

**Published:** 2025-05-31

**Authors:** Yohana Porto Calegari-Alves, Camila Innocente-Alves, Renata Pereira Costa, Aline Martins Faustino, Karyn Scheffler Schirma Farias, Mateus Boiani, Bruno Samuel Ardenghi Gonçalves, Marcio Dorn, Walter Orlando Beys-da-Silva, Lucélia Santi

**Affiliations:** 1 Laboratory of Molecular Microbiology and Proteomics, Faculty of Pharmacy, Federal University of Rio Grande do Sul, Porto Alegre 90610-000, RS, Brazil; calegari.yohanap@gmail.com (Y.P.C.-A.); camila.innocente@ufrgs.br (C.I.-A.); renatap.costaa@gmail.com (R.P.C.); alinemfaustino12@gmail.com (A.M.F.); karyn.schirma@gmail.com (K.S.S.F.); walter.beys@ufrgs.br (W.O.B.-d.-S.); 2 Institute of Informatics, Federal University of Rio Grande do Sul, Porto Alegre 91501-970, RS, Brazil; mboiani@inf.ufrgs.br (M.B.); bruno.samuel@ufrgs.br (B.S.A.G.); mdorn@inf.ufrgs.br (M.D.); 3 Center of Biotechnology, Federal University of Rio Grande do Sul, Porto Alegre 91501-970, RS, Brazil

**Keywords:** *Cryptococcus*, database, infection, host, yeast

## Abstract

Cryptococcosis is a serious fungal infection mainly caused by two *Cryptococcus* species, *Cryptococcus gattii* and *Cryptococcus neoformans* species complexes. Even though it is considered a dangerous disease, this infection is also neglected worldwide and its research is not adequately funded. The molecular understanding of the infection, which could help in the development of specific treatments and appropriate management, is hampered, as molecular data are not of easy access. With that purpose, our group developed a *Cryptococcus* molecular database, grouping published molecular data on gene and protein differential expression that occurred due to the infection. CRYPTOMICSDB presents a user-friendly interface, and users can search for both pathogen or host information and visualize data on experimental approaches, animal models or cell culture, *Cryptococcus* species and strain, genes and proteins. The database features 19,462 and 986,507 total genes related to the pathogen and host views, respectively. CRYPTOMICSDB is a powerful tool that can help health workers and microbiology researchers to better understand the molecular impact caused by cryptococcosis infection process.

## 1. Introduction

Cryptococcosis is a serious fungal infection caused by fungal pathogens of the *Cryptococcus gattii* and *Cryptococcus neoformans* species complexes. This infection is of great concern, especially in immunocompromised patients. For instance, *C. gattii* infection is commonly related to immunocompetent individuals whereas *C. neoformans* is related to immunocompromised patients [1]. Infection begins when an individual inhales fungal spores, and although the initial infection occurs in the pulmonary system, it can quickly spread to the brain via the hematogenous route and, if it crosses the blood–brain barrier (BBB), result in cryptococcal meningitis [2]. In fact, it is estimated 152,000 cases of cryptococcal meningitis occur in patients living with HIV worldwide [3].

Like many other fungal infections, cryptococcosis is a neglected disease in many countries, especially those with low incomes, resulting in greater difficulty in diagnosis and access to treatment, since first-line antifungals for the disease have a high cost [4]. In fact, it is one of the main causes of death among people living with HIV/AIDS, with a record of 112,000 deaths out of every 152,000 people infected [3]. However, immunocompetent individuals are also at risk of contracting the infection, mainly by the *C. gattii* species [5].

To address these gaps, molecular approaches such as transcriptomics and proteomics have been successfully applied to a better understanding of the infection process of several pathogens, including *Cryptococcus*. Differential expression of genes and proteins of pathogens and hosts after infection may be performed using these techniques to elucidate molecular mechanisms involved in host invasion, fungal adaptation and host response. *Cryptococcus* transmigration to the BBB may also be elucidated [6], as well as targets for therapy development [7]. Molecular approaches have also been applied to study the mechanism of action of potential antifungal therapies [8]. The fact that *Cryptococcus* is not a model organism restricts its molecular investigation. The substantial divergence of *C. neoformans* from other fungi [9] further impedes research by limiting the power of comparative analyses. In this scenario, molecular databases dedicated exclusively to pathogens, such as *Cryptococcus*, can help in understanding the molecular alterations caused by the yeast. In addition to CRYPTOMICSDB (Cryptococcus Omics Database), we created the ZIKA Virus Infection Database (ZIKAVID) (https://zikavid.org, accessed on 18 March 2025) after the Zika virus outbreak in Brazil and the SARS-CoV-2 infection database (SARSCOVIDB) (https://sarscovidb.org/, accessed on 18 March 2025) at the onset of the COVID-19 pandemic in 2020. Both databases are composed of genes and proteins that were found as altered after both infections in the scientific literature. 

Recognizing the limited molecular data available for *Cryptococcus* research and the absence of a dedicated database, we developed CRYPTOMICSDB to aid in the study of this complex disease. This database centralizes and organizes the large amounts of molecular data generated by transcriptomics and proteomics studies, allowing researchers to easily access and analyze gene and protein expression changes during infection of both *C. neoformans* and *C. gattii*. CRYPTOMICSDB provides a user-friendly interface to explore these changes from both the pathogen and the host perspectives, including data from *Homo sapiens* and *Mus musculus*. Users can also perform Gene Ontology (GO) and Kyoto Encyclopedia of Genes and Genomes (KEGG) pathway analyses. By centralizing this information, CRYPTOMICSDB aims to simplify the study of *Cryptococcus* infection mechanisms. By providing a comprehensive view of the molecular events involved in cryptococcosis, CRYPTOMICSDB can help researchers identify key pathways, potential drug targets, and biomarkers, ultimately accelerating the development of new diagnostic and therapeutic strategies. CRYPTOMICSDB is available at https://sbcb.inf.ufrgs.br/research/cryptomicsdb, accessed on 18 March 2025.

## 2. Materials and Methods

### 2.1. Selection of Published Scientific Literature

**Figure 1:**
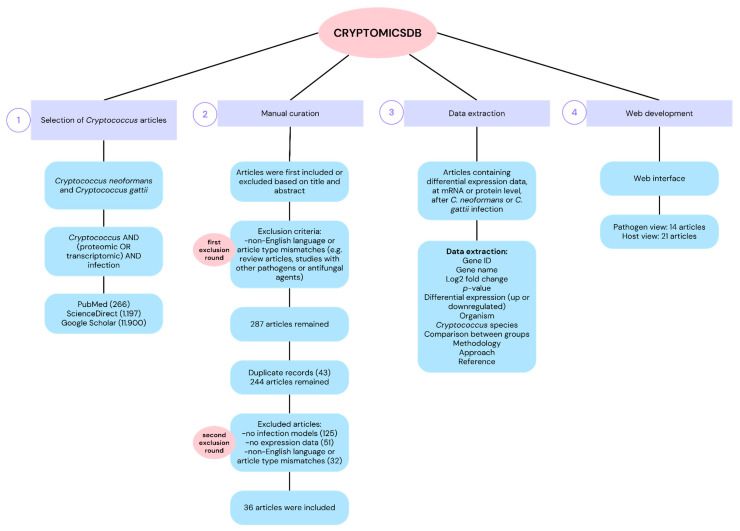
Flowchart of the construction process of CRYPTOMICSDB. (1) Systematic search for scientific articles on PubMed, ScienceDirect and Google Scholar using terms related to “*Cryptococcus*”, “infection”, “proteomic” and “transcriptomic”; (2) Inclusion/exclusion of articles through a manual curation by two independent researchers; (3) Extraction of differential expression data at mRNA and protein levels comprising information on gene/protein identification, differential expression type, host model, *Cryptococcus* strain, and molecular approach, among others; (4) Implementation of the database in a responsive web environment, featuring an interface for navigation through “pathogen view” (gene or protein with altered expression identified from *Cryptococcus* spp. after infection) and “host view” (gene or protein with altered expression in different hosts after *Cryptococcus* spp. infection).

The CRYPTOMICSDB was constructed through a systematic literature search conducted in the PubMed, ScienceDirect, and Google Scholar databases using the keywords “*Cryptococcus*”, “infection”, “proteomic”, and “transcriptomic”. The initial search retrieved 266 articles from Pubmed, 1,197 from ScienceDirect, and 11,900 from Google Scholar, published between January 2001 and May 2025. After manual curation, 287 articles remained. Duplicates were excluded, resulting in 244 unique articles. Each article was independently screened by two researchers based on the title and abstract. Inclusion criteria encompassed English-language studies involving proteomic or transcriptomic analyses of *Cryptococcus* infection with available differential expression data. Exclusion criteria included studies that did not involve an infection model or lacked molecular expression data. As a result, 208 articles were excluded—125 for not involving infection models, 51 for insufficient expression data, and 32 for language or article type mismatches (i.e., review articles or other pathogens). Ultimately, 36 articles were selected for data extraction, comprising 14 studies with pathogen-derived data and 21 with host-derived data (Supplementary Table S1). The complete construction workflow of the CRYPTOMICSDB is summarized in Figure 1.

### 2.2. Data Extraction

For each selected article, molecular data were manually extracted by two independent curators (see Table 1). Extracted variables included gene/protein identifier and name, log2 fold change, *p*-value, expression direction (up- or down-regulated), organism, *Cryptococcus* species, sample comparison groups, methodological category (transcriptomics or proteomics), and specific technique. When duplicate genes or proteins were reported across multiple studies, each instance was retained along with its original reference to preserve data traceability. Gene identifiers and protein names were harmonized across different databases (e.g., UniProt, NCBI) to standardize entries. In studies lacking explicit statistical thresholds, a default significance criterion of *p* < 0.05 was adopted. Discrepancies between curators were resolved by consensus during quality control rounds.

### 2.3. Gene Ontology Analysis

The list of genes and proteins collected during data curation was submitted to GO Enrichment Analysis using the online tool called “Database for Annotation, Visualization, and Integrated Discovery” (DAVID, available at https://davidbioinformatics.nih.gov/, accessed on 26 March 2025). Molecular functions, biological processes, cellular components, and metabolic pathways (KEGG) were assigned to this list, and only those that were statistically significant (*p* value < 0.05) were considered.

### 2.4. Webpage Construction

The CRYPTOMICSDB web application was architected utilizing a decoupled client–server architecture, delineating distinct frontend and backend components. The client-side interface was developed using SvelteKit 2.0.0, employing both JavaScript (version ES14) and TypeScript (version 5.8.3) to implement dynamic functionalities and enforce static type checking. Tailwind CSS was integrated to facilitate a responsive and aesthetically consistent user experience. This frontend component communicates with the server-side via HTTP requests directed to a dedicated Application Programming Interface (API). The server-side, implemented with Python 3.10 and FastAPI version 0.115.0, functions as the intermediary layer, processing data requests and generating corresponding responses. DuckDB was selected as the Database Management System (DBMS) to enable efficient creation and maintenance of the curated CRYPTOMICSDB. HTTP routes, defined within the FastAPI application, provide the requisite endpoints for accessing and retrieving data entries from the DuckDB database, thereby ensuring seamless data exchange between the client interface and the persistent data storage.

## 3. Results and Discussion

Beyond compiling molecular data related to cryptococcal infections, CRYPTOMICSDB offers practical applications that support both basic and translational research. Its dual interface—encompassing both pathogen and host perspectives—allows researchers to explore gene and protein expression changes under various experimental conditions. For example, researchers studying the mechanisms by which *Cryptococcus* crosses the blood–brain barrier can retrieve pathogenic genes overexpressed during CNS infection and identify their associated biological processes using integrated GO annotations. Similarly, users interested in host immune responses can access data from samples from their host of interest and identify key pathways affected by infection, such as cytokine signaling or oxidative stress. These functionalities enable the generation of biologically relevant hypotheses, the identification of potential therapeutic targets, and the validation of experimental results against published omics data. Thus, CRYPTOMICSDB is not only a repository of curated information, but also a robust exploratory tool that fills gaps in research on cryptococcal pathogenesis.

**Figure 2:**
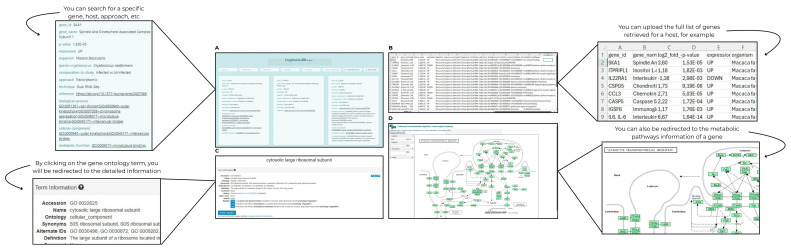
Overview of the search functionalities in CRYPTOMICSDB. (**A**) Filtering entries by organism (e.g., *Macaca fascicularis*); (**B**) Exporting results in CSV format; (**C**) Example of gene ontology for term definitions; (**D**) Example of KEGG pathway visualization.

For example, CRYPTOMICSDB allows users to filter entries by specifying an “organism” in the search field (as shown in Figure 2A). The resulting data can then be downloaded in CSV format for further analysis (Figure 2B). Users can also perform targeted searches using specific “gene_id” or “gene_name” identifiers. Clicking on a Gene Ontology (GO) term (e.g., “biological process,” “cellular component,” or “molecular function”) will redirect the user to the corresponding GO database entry, providing a comprehensive definition and related information (Figure 2C). Similarly, selecting a metabolic pathway links to the KEGG pathways database, enabling users to visualize the complete pathway map and understand its biological significance (Figure 2D). Furthermore, searches can be refined by methodology (e.g., transcriptomics or proteomics) or by experimental approach (e.g., RNA-seq or MudPIT). These versatile search functions enable researchers to tailor their database exploration to their specific research interests, facilitating hypothesis generation, data comparison, and validation of findings in the literature.

Due to its status as a neglected disease, cryptococcosis research receives limited funding, estimated at less than 0.5% worldwide [10,11]. Consequently, a critical lack of gene and protein expression data impedes molecular studies and our understanding of the infection. CRYPTOMICSDB was created to bridge this information gap by compiling available gene and protein expression data from published research.

**Figure 3:**
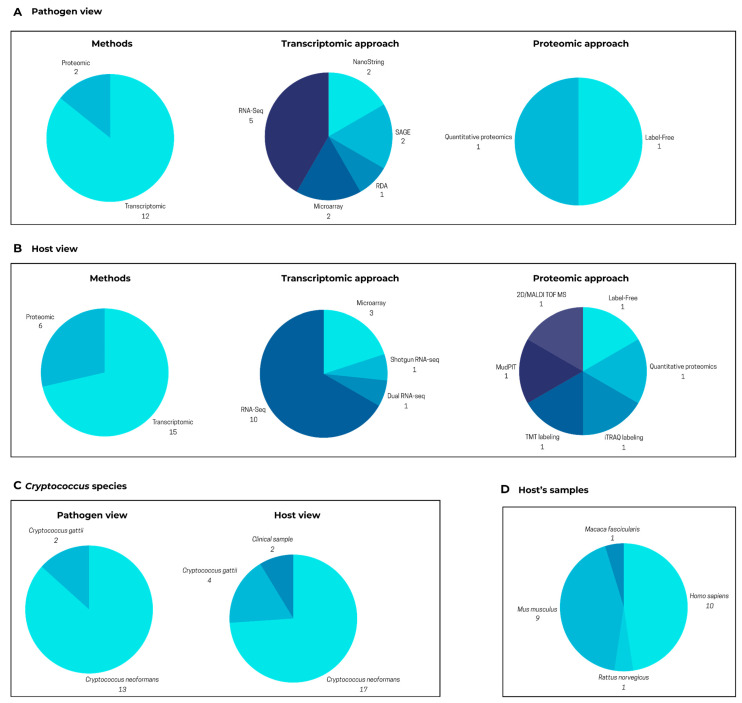
Summary of content in CRYPTOMICSDB. (**A**) Pathogen view: number of articles reporting gene or protein with altered expression identified in *Cryptococcus* spp. following infection. (**B**) Host view: number or articles reporting gene or protein with altered expression identified in various host organisms following *Cryptococcus* spp. infection. Charts show a number of articles identified for each subject. (**C**) Distribution of included studies based on the *Cryptococcus* species (*C. neoformans* or *C. gattii*) analyzed in the pathogen and host views. Some articles examined both species and were counted in each category. (**D**) Host organisms and sample types in the included studies. The pathogen view presents molecular data from *Cryptococcus*, while the host view presents data from organisms infected by *Cryptococcus* (e.g., *Homo sapiens*, *Mus musculus*).

In CRYPTOMICSDB, users can visualize data connecting a pathogen and its various hosts. Currently, the database’s pathogen view contains a total of 19,462 genes (5863 of which are unique), while the host view encompasses 986,507 total genes (39,982 unique). These data have been compiled from 14 and 21 published research articles for the pathogen and hosts, respectively. Figure 3A,B illustrate that these studies employed diverse methodologies, with transcriptomics being the most frequently utilized approach for molecular-level investigations.

Among the retrieved results, the studied *Cryptococcus* species included *C. gattii* and *C. neoformans*. From the pathogen perspective, *C. gattii* was examined in two articles, whereas *C. neoformans* appeared in 13 (Figure 3C). From the host perspective, *C. gattii* was the focus of four articles, while *C. neoformans* was analyzed in 17 (Figure 3C). Additionally, two studies utilizing clinical samples did not specify the *Cryptococcus* species, hampering the detailed understanding of the infection process, as these two species affect distinct populations. Unlike typical databases that solely focus on the pathogen, CRYPTOMICSDB integrates data about various hosts of *Cryptococcus*, enabling a more comprehensive understanding of the infection process. On the homepage, researchers can specifically select either a “host view” or a “pathogen view” and even search for particular organisms.

The host samples integrated into CRYPTOMICSDB originate from the following organisms, based on the number of contributing research articles (as shown in Figure 3D): *Homo sapiens* (10 articles), *Mus musculus* (9 articles), *Rattus norvegicus* (1 article), and *Macaca fascicularis* (1 article). Notably, while numerous studies within the database employed cell lines (six human, six mouse) and lung or brain tissues (two mouse, one rat, one combined mouse and monkey, and two human), a limited number of investigations (only three) analyzed blood samples (one mouse, two from human patients with cryptococcal meningitis). This scarcity of research using primary human samples, such as blood or cerebrospinal fluid, underscores a significant gap in our understanding of the infection mechanism in humans. Future studies analyzing samples from both immunocompromised individuals infected with *C. neoformans* and immunocompetent individuals infected with *C. gattii* would definitely provide valuable insights into this mechanism.

Since CRYPTOMICSDB presents data on differential expression of genes and proteins, it is crucial that the articles present detailed information on statistical analysis involved in the dysregulation type. However, we faced challenges regarding the lack of this type of information, and we adopted *p* < 0.05 in these cases. Information was added to the database as presented in the articles (up or downregulated), but with no fold change value. This information is essential for a proper understanding of the infection process. Another challenge was the lack of standardization in gene IDs and names. Many articles relied on different gene and protein databases, such as UniProt and NCBI, which hindered standardization and, consequently, data categorization.

To date, available databases with gene and protein expression information on yeasts include *Saccharomyces cerevisiae* (https://www.yeastgenome.org/, accessed on 18 March 2025), *Pichia pastoris* (http://pichiagenome-ext.boku.ac.at/, accessed on 18 March 2025), and *Candida albicans* (http://www.candidagenome.org/, accessed on 18 March 2025). For the *Cryptococcus* genus, our search identified only one relevant database: the *C. neoformans* Phenome Gateway (at http://www.cryptococcus.org/, accessed on 18 March 2025). However, this resource is limited to a single species (*C. neoformans*) and primarily focuses on transcription factors, kinases, and phosphatases. To the best of our knowledge, CRYPTOMICSDB is the first *Cryptococcus* database that englobes the etiological agents of cryptococcosis: *C. neoformans* and *C. gattii* species complexes. In addition, as a differential, our database also integrates molecular data on different hosts infected by both fungi, contributing to the elucidation of the infection process in different organisms.

## 4. Conclusions

CRYPTOMICSDB is a newly described database that englobes molecular data related to *C. gattii* and *C. neoformans* experiments, presenting altered genes and proteins in both pathogen and host views due to the infection. Data can be used to perform bioinformatic analysis of different experiments, creating and corroborating with researchers’ hypotheses. Also, due to its user-friendly interface, no previous experience in bioinformatic is required.

## Figures and Tables

**Table 1:** Description of the information grouped in CRYPTOMICSDB.

Gene ID	Gene ID According to UniProt
Gene or protein name	Official gene or protein name for each entry
Log2 fold change	Criteria applied to define as differentially expressed gene or protein
*p*-value	Statistical criteria applied to define as differentially expressed gene or protein
Organism	Organism described as a sample in the study, for example, *Mus musculus*
*Cryptococcus* species	Complex of species causing infection: *Cryptococcus neoformans* or *Cryptococcus gattii*
Differential expression	Gene or protein expression according to the parameters described in the study
Comparison between groups	For example, comparing infected vs. control groups
Method	Transcriptomics or proteomics
Approach	Technique applied according to the method described in the paper

## Data Availability

The original contributions presented in this study are included in the article. Further inquiries can be directed to the corresponding author.

## Supplementary Materials

The following supporting information can be downloaded at: https://www.mdpi.com/article/10.3390/jof11060425/s1, Table S1: List of Curated Articles Used for Host and Pathogen Data Compilation in CRYPTOMICSDB.
